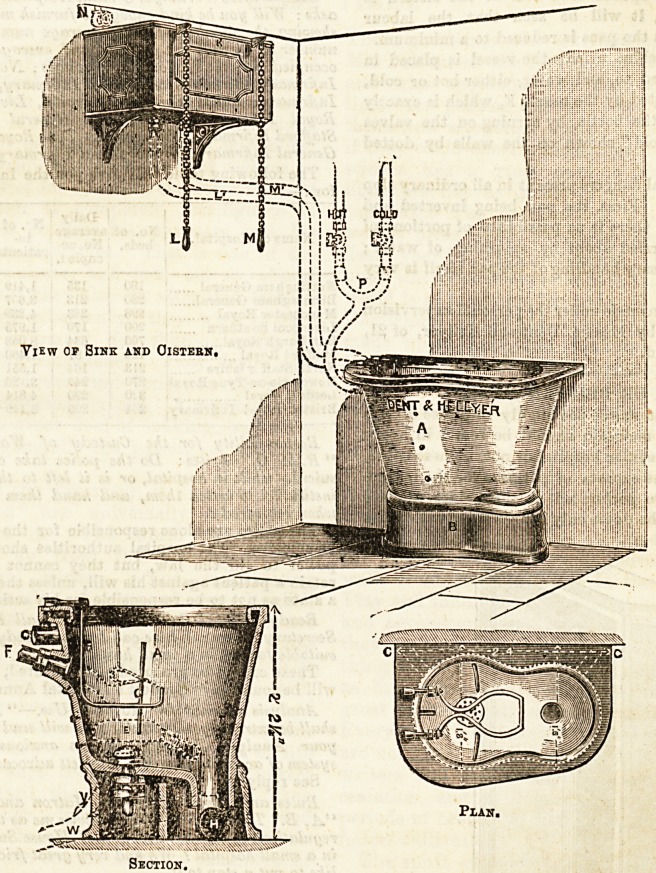# A New Form of Hospital Slop Sink

**Published:** 1892-05-14

**Authors:** 


### A NEW FORM OF HOSPITAL SLOP SINK.

The operation of emptying and cleansing bed pans is one which involves no light risk to the nurse whose duty it is to perform it, and necessitates the most scrupulous care on her part. The risk becomes a positive and serious danger when the excreta of typhoid fever have to be dealt with, and the neglect of due precaution may lead to fatal results. It is with the object of minimising the risk, and, if possible, protecting a nurse from the results of her own carelessness that the slop sink, of which we give an illustration to-day, has been designed. The sink, which is called the "McHardy sink" after its inventor, the well-known professor of ophthalmology in King's College, London, is made throughout of glazed fire-clay.

View of Sink and Cistern.

Section.

Plan.

In the section the position of the bed-pan when it is placed in the sink is indicated by the dotted lines enclosing the letter E. Just above the bed-pan is another set of dotted lines marked C with an L shaped arrangement suspended from the top. C represents a urine bottle, and the L represents a cradle in which the bottle is supported. The cradle is removable and would only be fixed on when a urine bottle had to be cleaned out.

In emptying a bed pan, the pan being held by the spout is turned over with the opening downwards, and rests in the position indicated on the section, being steadied by three strips of india-rubber, one at the back and one at each side. One of these strips is shown at A in the section, and all three are shown in the plan. Immediately under the centre of the bed-pan is a perforated jet D, fed with cold water from the cistern (K, in view). By pulling the handle L a jet of water is projected upwards into the pan, of sufficient quantity and force to wash the contents out through the neck, and down into the waste-pipe H, the pan itself the while being so firmly held in position by the rubber strips that not a drop of water escapes, and the possibility of the nurse being splashed is entirely avoided. The handle M works the valve by which water is admitted to the rim of the sink and the sides flushed down. As both these handles can be pulled simultaneously, and as the valves once opened each half of the cistern is emptied automatically, it will be seen that the labour involved in cleaning out the pans is reduced to a minimum.

To clean out glass or china urinals the vessel is placed in the cradle before referred to, and water, either hot or cold, or both mixed, is admitted by the nozzle F, which is exactly opposite the mouth of the bottle, by turning on the valves marked "hot" and "cold" shown on the walls by dotted lines.

Thus the two principal dangers present in all ordinary slop sinks are here avoided. First, the pan being inverted and flushed out from below, there is no possibility of portions of fæcal matter being carried about by splashing of water; and, secondly, the necessary handling of the pan itself is very materially reduced.

The slop-sink has been made under the personal supervision of Professor McHardy, by Messrs. Dent and Hellyer, of 21, Newcastle Street, Strand.

## Figures and Tables

**Figure f1:**